# The Association of Social Emotions, Perceived Efficiency, Transparency of the Government, Concerns about COVID-19, and Confidence in Fighting the Pandemic under the Week-Long Lockdown in Shenzhen, China

**DOI:** 10.3390/ijerph191811173

**Published:** 2022-09-06

**Authors:** Xiaozhe Peng, Jiajun Huang, Kaixin Liang, Xinli Chi

**Affiliations:** 1 School of Psychology, Shenzhen University, Shenzhen 518060, China; 2 The Shenzhen Humanities & Social Sciences Key Research Bases, Center for Mental Health, Shenzhen University, Shenzhen 518060, China

**Keywords:** social emotions, concerns about COVID-19, perceived efficiency and transparency of the government, confidence in fighting COVID-19

## Abstract

The outbreak and spread of the COVID-19 pandemic have had a far-reaching impact. The present study investigated the factors primarily affecting the execution of the control measures, including social emotions, concerns about the pandemic, perceived efficiency, transparency of the government in publishing the pandemic-related information, and confidence in fighting the pandemic. Specifically, we examined the differences in these factors across four areas (i.e., lockdown area, control area, prevention area, and safe area) according to different COVID control measures under the week-long lockdown in Shenzhen. We found that social emotions, concerns about the pandemic, perceived efficiency of the government, and confidence in fighting the pandemic were more negative in the lockdown area than that in other areas. More importantly, after controlling for areas and education level of participants, the emotion of optimism, concerns about the COVID-19 pandemic, perceived efficiency, and perceived transparency of the government in releasing COVID-19 relevant information positively predicted confidence in fighting the pandemic, while anger negatively predicted confidence in fighting the pandemic. Therefore, the government and communities could make efforts at effective communication and find innovative approaches to make individuals (especially in the lockdown area) maintain social connections, reduce negative emotions, and enhance confidence in combating the pandemic.

## 1. Introduction

The outbreak and spread of the COVID-19 pandemic have had a far-reaching impact. The fast spread and increased mortality of COVID-19 prompted the government to implement different control and prevention measures, such as social distancing, face mask use, and vaccination. The execution of these measures has been central in fighting COVID-19 and has been associated with the public’s emotions, perceptions, and attitudes toward the government, and confidence in fighting the pandemic [1,2]. First, in terms of emotions, there is a dilemma that though harsh restrictions such as lockdown can reduce the transmission and mortality rate by reducing the frequency of physical contact and social connections, they will increase negative emotions [3]. Specifically, social connections can relieve loneliness, depression, and other negative emotions which might result from social distancing efforts to fight the pandemic [4]. Therefore, as a primary factor to influence the implementation of pandemic prevention and control measures [5,6], citizens’ emotions associated with social isolation should be considered when the government imposes control measures.

Specifically, negative emotions such as anger and fear were mainly found in a study examining the public emotions expressed on social media toward social distancing measures during COVID-19 [7]. It was suggested governments respond swiftly to relieve residents’ anxiety, stress, and other negative emotions during the pandemic [6,8]. Correspondingly, a study found that positive emotions (e.g., optimism, calm, or hope) can support people going through the COVID-19 pandemic [9,10]. Studies also suggest that stringent restrictions such as global lockdown affected people’s emotions, and, in the long term, will affect mental health and well-being [11,12,13]. However, what the differences were in residents’ emotions across areas imposed with different control measures in a city remained unclear.

Second, people’s perceptions, attitudes toward the government, and their confidence in fighting the pandemic were suggested to be associated with the perceived efficacy and implementation of the control measures [5,14]. In terms of public perceptions and attitudes toward the government, studies found that perception about the capability of the government impacted the perceived efficacy and the execution of the control measures [5,15]. People who trust their government are more likely to adhere to the control measures, such as responding positively to vaccination [5]. Specifically, effective communication of the government, including transparent information about public events and swift responses to people’s requirements, can enhance public confidence to combat the pandemic [16,17,18]. Considering social emotions regarding concerns about the pandemic, on the one hand, perceived transparent and effective communication by the government is associated with a higher evaluation of the efficacy of control restrictions and adherence to health measures [5]. On the other hand, transparent and effective communication about the efficiency of the introduced restrictions can also reduce negative emotions [1]. As far as confidence is concerned, studies have found that confidence in fighting COVID-19 influences the implementation of control measures and further influences the effect of the policies to fight the pandemic [17,19]. Besides emotions, people’s perceived efficacy of control and prevention measures, their adherence to control measures, and their attitude toward the government (such as satisfaction with the government, trust in the government, and perceptions of the responses of the government) are important factors associated with confidence in fighting the pandemic [1,5,20,21,22]. Previous studies investigating confidence to combat the pandemic suggested that the perceived efficacy of control measures, such as COVID-19 vaccination, and quarantine will affect the execution of these measures, accordingly affecting residents’ beliefs in controlling the pandemic [6,23,24].

According to the studies mentioned above, social emotions, perceptions of government capability, and confidence in fighting the pandemic were influenced by the control measures. Understanding these factors can help to inform the government and authorities to follow effective and efficient communication approaches to affect residents’ support and adherence to the control measures. In the present study, people’s perceived transparency and perceived efficiency of the government in publishing pandemic-relevant information were used to represent the effective communication of the government. Furthermore, a recent study found that concerns regarding pandemic information positively predicted the public’s worry and social emotions, which in turn predicted a supportive attitude toward the prevention measures [25]. Thus, we assume that with the perception of transparency and efficiency of published pandemic-related information by the government, concerns about the pandemic can predict confidence in fighting COVID-19.

However, few studies compared these factors among different areas with different control measures, while in the present study, we investigated these factors simultaneously in the same city. More specifically, on 13 March 2022, Shenzhen (a city bordering Hongkong and a hub of the technical sector of China) identified cases of the Omicron variant COVID-19. To prevent a further outbreak of the pandemic, the Shenzhen government stated and performed a series of control measures. During the full-day lockdown, to make pandemic prevention and control more targeted, districts were divided by the severity of the control: the lockdown area, control area, prevention area, and safe area. According to the statement of the Shenzhen Government online, there were different social connections such as “residents in lockdown areas must stay at home and are provided with door-to-door delivery service, residents in control areas must stay at home for the first four days after the restrictions are imposed, with door-to-door delivery service provided. After risk assessment, citizens must remain in their residential buildings and can pick up their parcels at staggered hours from the fifth day to the end of the restrictions…. while in prevention areas, gatherings should be avoided and residents should conduct self-health monitoring within a week” according to the criteria for lifting COVID-19 restrictions imposed by Shenzhen government [26]. Accordingly, Shenzhen experienced full days under lockdown from 13 March 2022 to 20 March 2022. During the lockdown, people had to stay at home, public transport was suspended, non-essential businesses were closed, and daily mass nucleic acid testing was performed. From 17 March, five administrative districts (out of the central business) in Shenzhen achieved “community-wide COVID-19 dynamic clearance” [27]. From 20 March, most of the center businesses reopened, and a majority of people in these districts could restart work. Public transport also resumed from 18 March. However, certain areas (e.g., the southern part of Futian District) still needed to have control measures.

The present study investigated the differences in social emotions, concerns about the COVID-19 pandemic, perceived efficiency of the government, and confidence in fighting the COVID-19 pandemic across four areas according to differentiated COVID control measures. More importantly, the present study investigated the key factors predicting confidence in fighting COVID-19. As governmental anti-COVID-19 control measures are ongoing, it is crucial to have insight into residents’ emotions to reduce negative emotions, enhance governmental efforts on publishing pandemic-relevant information, respond swiftly to the pandemic situation to build regulations, and make decisions in the best interests of the public, thus promoting confidence combating the pandemic [28]. Based on these findings, the present study formulated two research questions (RQs) and two hypotheses:

RQ1: What were the differences in social emotions, concerns about the pandemic, perceived efficiency, and transparency in releasing COVID-19 relevant information across different areas (i.e., lockdown area, control area, prevention area, and safe area) during the Shenzhen one-week lockdown?

RQ2: To what extent could social emotions, concerns about the pandemic, efficiency, and transparency in relation of COVID-19 relevant information mentioned above predict confidence in fighting the pandemic?

**Hypothesis** **1.** *We expected that in the lockdown and control areas, negative emotions would increase while the following would decrease: positive emotions, concerns about the pandemic, perceived transparency of the government in releasing COVID-19 relevant information, positive expectations about the development of the pandemic in the coming month, and confidence fighting the pandemic.*

**Hypothesis** **2.** *Moreover, positive emotions, concerns about the COVID-19 pandemic, perceived efficiency, and perceived transparency of the government in releasing COVID-19 relevant information were assumed to positively predict confidence in fighting the COVID-19 pandemic.*

Considering methodology, survey self-reports were commonly used in research investigating social emotions and attitudes [29]. Compared with interviews, observations, and other behavioral measures, the advantages of self-report included that it can be utilized relatively quickly and promote truthful responses since it can be performed in private [30]. Specifically, online survey was widely used in studies examining public mental health and other psychological reactions during the COVID-19 pandemic [31]. Thus, survey was utilized in the present study.

## 2. Survey and Methods

### 2.1. Procedure

From 21 to 23 March 2022, a snowball sampling strategy was used. Specifically, participants were recruited by disseminating the recruitment advertisements on Wechat (a popular social media platform in China) in broadcasting using the public account of our lab, or narrow-casting by sharing this advertisement to Wechat moments, and to residents’ communication groups of different areas. Eight types of recruitment advertisements were randomly disseminated, with each advertisement including 3 Wechat accounts of 24 trained research assistants. Thus, people who wanted to join this research can randomly choose 1 of the 3 Wechat accounts and add this research assistant as a “friend”. Then the research assistant sent the link of an anonymous online survey to the participant. Research assistants were asked not to provide any additional information except send the link of the survey to participants.

Participants had to complete an online survey that consisted of an introduction of the study, informed statements, and questions through an online survey platform (Tencent Survey). Both in the “participants recruitment notice” and introduction of the survey, we introduced this research and mentioned that we only recruit Shenzhen residents who at least lived in Shenzhen from 13 March 2022. To confirm if participants lived in Shenzhen, the initial question on the survey asked participants whether they lived in Shenzhen from 13 March 2022. The survey would be terminated, and participants would be thanked if they selected “not living in Shenzhen from 13 March 2022”. Participants proceeded to the next survey question when they agreed to participate in the study and submitted the informed consent online. Ten days after participants finished the survey (and our team checked whether they finished the survey carelessly, see the following for details), appropriate reimbursement was paid (through Wechat pay) for participation.

### 2.2. Participants

A total of 1726 participants took part in this study. Data from 76 participants were excluded from the analyses due to them not living in Shenzhen, and data from 15 participants were excluded because they filled out the survey carelessly (finished the survey within 60 s), leaving a final sample of 1635 participants. Descriptions are shown in Table 1. The present study was approved by the institutional review board of Shenzhen University.

### 2.3. Survey

In this survey, participants were asked to answer the following questions:

#### 2.3.1. Social Emotions

Q1. “Please rate the following emotions you felt during the very last week?” Participants rated their feelings about “optimism/calm/worry/helplessness/fear/sadness/anger/restlessness” by a 5-point Likert rating scale.

#### 2.3.2. Concerns about the Pandemic

Q2. “Please choose the time you spent each day reading or searching information about COVID-19 on the internet, please note that access to the internet includes using Wechat or Sina Weblog by computer, cellphone, etc.” Participants chose one from the options “less than 30 min”, “30 to 60 min”, “1 to 3 h”, “3 to 5 h”, “more than 5 h”.

Q3. “How concerned are you about the information regarding COVID-19?”. Participants had to rate from 1 (“very low”) to 4 (“very high”).

#### 2.3.3. Perceived Efficiency and Transparency of the Government

Q4. “How do you rate the efficiency of the Shenzhen government in publishing the information about COVID-19?” Participants had to rate from 1 (“very low”) to 4 (“very high”).

Q5. “How do you rate the transparency of the Shenzhen government in publishing the information about COVID-19?” Participants had to rate from 1 (“low”) to 3 (“high”).

#### 2.3.4. Confidence in Fighting the Pandemic

Q6. “How do you expect COVID-19 will develop in the coming month?” Participants had to rate from 1 (“much more severe”) to 4 (“much more relief”).

Q7. “How confident are you in the fight against COVD-19 in Shenzhen?” Participants had to rate from 1 (“very negative”) to 4 (“very positive”).

## 3. Data Analysis

As an effective tool, and the most widely used software for quantitative data analysis, SPSS Statistics 26.0 (IBM, Somers, NY, USA) was used for statistical analysis in the current study [32,33]. A two-way repeated-measures ANOVA was performed, with emotion (optimism vs. calm vs. worry vs. helplessness vs. fear vs. sadness vs. anger vs. restlessness) as a within-subject variable, and area (lockdown area vs. control area vs. prevention area vs. safe area) as a between-subject variable. Descriptive data are shown as mean ± standard error. The Greenhouse–Geisser is frequently used to assess changes in results with observations in the study with within-subjects. When the assumption of sphericity is violated in statistical analysis, the Greenhouse–Geisser correction is robust to the violation [34]. Thus, current results of Greenhouse–Geisser correction were reported if Mauchly’s test of sphericity was significant. Moreover, Bonferroni correction is widely used to adjust *p* values to avoid type I error when making multiple comparisons [35]. Thus, Bonferroni correction was used in all pairwise multiple comparisons in the present ANOVA.

A one-way ANOVA was performed to test the differences of concerns about the pandemic, perceived efficiency of the government, perceived transparency of the government in publishing the pandemic-related information, and confidence in fighting the pandemic among different areas, respectively. Bonferroni correction was used in all pairwise multiple comparisons in ANOVA.

Hierarchical regression analyses were conducted to assess whether the areas, emotions, concerns about the pandemic, perceived efficiency of the government, and perceived transparency of the government in publishing the pandemic-related information can predict confidence in fighting the pandemic (the average of Q6 and Q7 in the survey). Variables in individual characteristics, including areas and education level, were entered into Model 1, followed by the variables in social emotions (such as optimism, calm, fear, anger) were entered into Model 2. Concerns about the pandemic, perceived efficiency and transparency of the government were entered into Model 3. Statistical significance was considered in relation to an alpha of 0.05 in all analyses (two-tailed). Variance inflation factors (VIF) indicated that there was no multicollinearity between independent variables (all predictors showed VIF < 2.5).

## 4. Results

### 4.1. Descriptive Analysis

As shown in Table 1, of 1635 participants, 1071 (65.5%) were females. A total of 9 participants (0.6%) had a primary school degree, 42 (2.6%) had a middle school or technical school degree, 112 (6.9%) had a high school degree, 314 (19.2%) had a college degree, 964 (59%) had an undergraduate or bachelor’s degree, 194 (11.9%) had a master’s degree or above. Regarding regional description, 111 (6.8%) participants came from the lockdown area, 321 (19.6%) were from the control area, 648 (39.6%) were from the prevention area, and 555 (33.9%) were from the safe area. Participants reported the following ages: 1106 (67.6%) 18–29, 357 (21.8%) 30–39, 116 (7.1%) 40–49, 49 (3%) 50–59, and 7 (0.4%) 60 or older.

### 4.2. Social Emotions

Across all participants, the average rating of positive emotions was 3.34 (*SE* = 0.02), including optimism (3.34 ± 0.02) and calm (3.35 ± 0.03). The average rating of negative emotions was 2.22 (*SE* = 0.02), including worry (2.9 ± 0.03), helplessness (2.11 ± 0.03), fear (1.92 ± 0.03), sadness (2.11 ± 0.03), anger (1.89 ± 0.03), and restlessness (2.36 ± 0.03) (Table 2).

**Figure 1:**
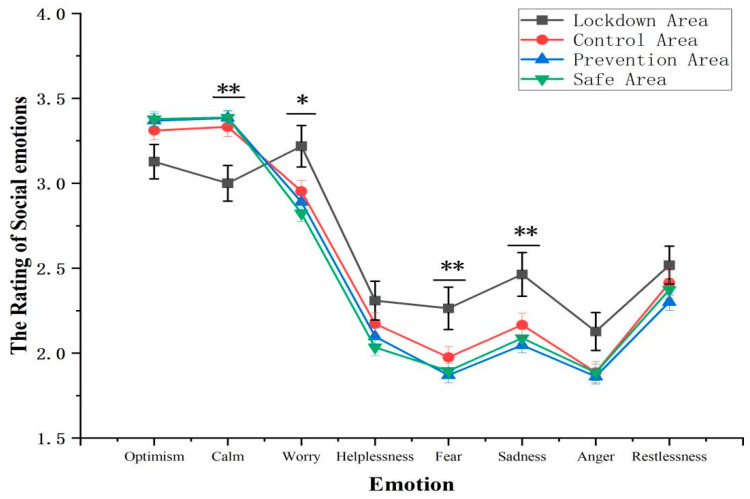
Social emotions in different areas. * *p* < 0.05, ** *p* < 0.01.

As shown in Figure 1, the interaction between emotion and areas was significant, *F* (10.053, 5465.395) = 3.45, *p* < 0.001, *η*^2^*_p_* = 0.006. Specifically, the simple effect of calm was significant, *F* (3, 1631) = 5.44, *p* < 0.01, *η*^2^*_p_* = 0.010. Participants who were in the lockdown area reported significantly lower rating of calm (2.98 ± 0.11) compared with that in the control area (3.33 ± 0.06), the prevention area (3.39 ± 0.04), and the safe area (3.39 ± 0.04). The simple effect of worry was significant, *F* (3, 1631) = 3.48, *p* < 0.05, *η*^2^*_p_* = 0.006. Participants living in the lockdown area reported a significantly higher rating of worry (3.21 ± 0.12) compared with that in the safe area (2.82 ± 0.05). The simple effect of fear was significant, *F* (3, 1631) = 3.93, *p* < 0.01, *η*^2^*_p_* = 0.007. Participants living in the lockdown area reported a significantly higher rating of fear (2.25 ± 0.12) compared with that in the prevention area (1.87 ± 0.04) and the safe area (1.90 ± 0.05). The simple effect of sadness was significant, *F* (3, 1631) = 4.05, *p* < 0.01, *η*^2^*_p_* = 0.007. Participants living in the lockdown area reported a significantly higher rating of sadness (2.45 ± 0.13) compared with that in the prevention area (2.05 ± 0.04) and the safe area (2.09 ± 0.05). In contrast, the simple effect of optimism was marginally significant, *F* (3, 1631) = 2.58, *p* = 0.052, *η*^2^*_p_* = 0.005. The rating of optimism was highest in the safe area (3.38 ± 0.04), followed by that in the prevention area (3.37 ± 0.04), the control area (3.3 ± 0.06), and the lockdown area (3.11 ± 0.10). The simple effect of helplessness, anger, and restlessness were not significant, all *ps* > 0.1.

### 4.3. Concerns about the Pandemic

**Figure 2:**
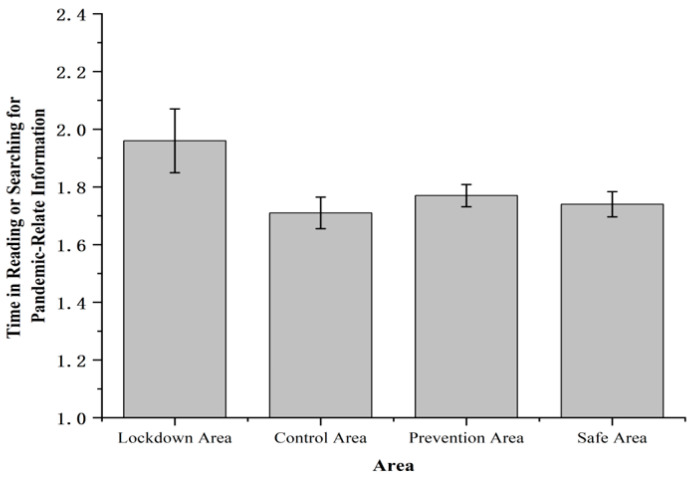
Time in reading or searching for pandemic-related information. Note: The rating of time in reading or searching for pandemic-related information was measured on a 5-point scale (1 = less than 30 min, 2 = 30 to 60 min, 3 = 1 to 3 h, 4 = 3 to 5 h, 5 = more than 5 h).

There were no significant differences in the time reading or searching for pandemic-related information among four areas, *F* (3, 1631) = 1.78, *p* > 0.1, *η*^2^*_p_* = 0.003 (Figure 2).

**Figure 3:**
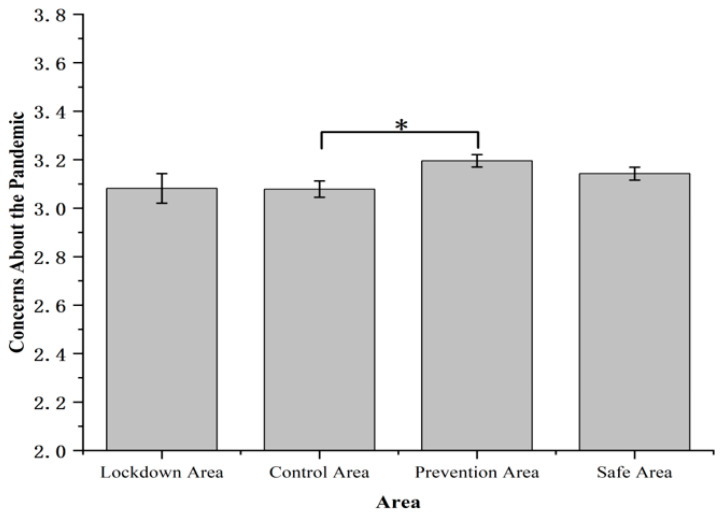
Concerns about the pandemic. Note: The rating of concerns about the pandemic was measured on a 4-point scale (1 = not at all, 2 = a little, 3 = a lot, 4 = very much). Error bars represent standard errors. * *p* < 0.05.

Concerns about the pandemic information were significantly different among four areas, *F* (3, 1631) = 2.97, *p* < 0.05, *η*^2^*_p_* = 0.005 (Figure 3). Participants in the control area (3.08 ± 0.03) reported significantly lower concerns about the pandemic compared with that in the prevention area (3.20 ± 0.03), *p* < 0.05.

### 4.4. Perceived Efficiency and Transparency of the Government

**Figure 4:**
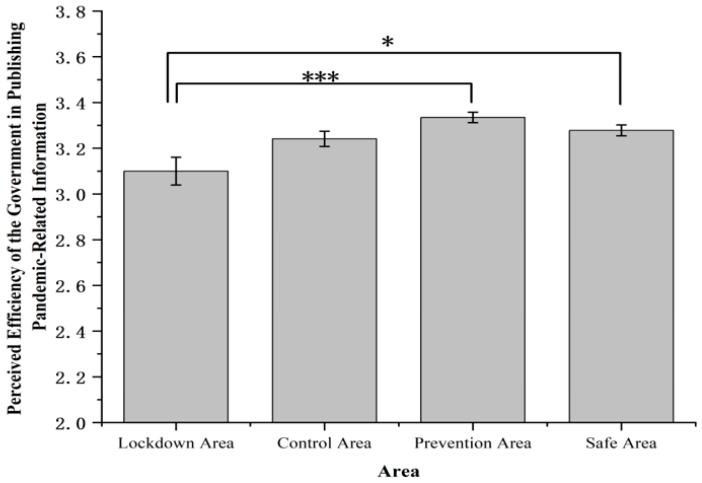
Perceived efficiency of the government in publishing pandemic-related information. Note: The rating of the efficiency of the government in publishing pandemic-related information was measured on a 4-point scale (1 = very slow, 2 = slow, 3 = high, 4 = very high). Error bars represent standard errors. * *p* < 0.05, *** *p* < 0.001.

Perceived efficiency of the government in publishing pandemic-related information showed significant differences among four areas, *F* (3, 1631) = 6.07, *p* < 0.001, *η*^2^*_p_* = 0.011 (Figure 4). Participants in the lockdown area (3.10 ± 0.06) reported lower rating of efficiency of the government compared with the prevention area (3.34 ± 0.02, *p* < 0.001) and the safe area (3.28 ± 0.02, *p* < 0.05).

**Figure 5:**
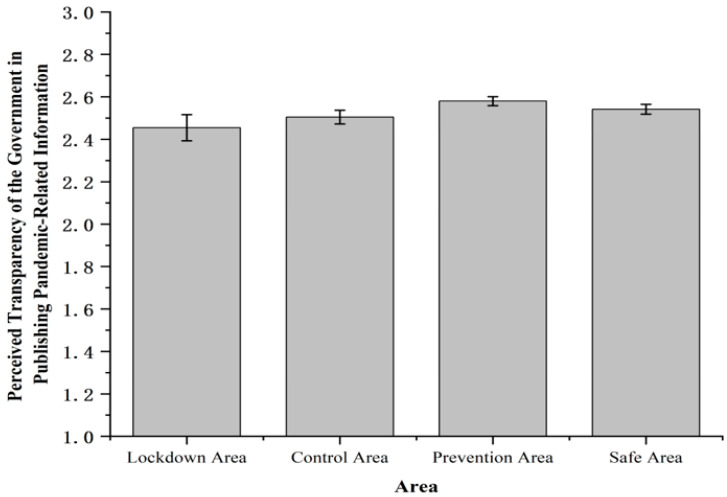
Perceived transparency of the government in publishing pandemic-related information. Note: The rating of the transparency of the government in publishing pandemic-related information was measured on a 3-point scale (1 = low, 2 = medium, 3 = high). Error bars represent standard errors.

Otherwise, we found that the differences of the perceptions of the transparency of public information on the pandemic among four areas were marginally significant, *F* (3, 1631) = 2.52, *p* = 0.056, *η*^2^*_p_* = 0.005 (Figure 5). The rating of the transparency of public information on the pandemic was highest in the prevention area (2.58 ± 0.02), followed by the safe area (2.54 ± 0.02), the control area (2.50 ± 0.03), and the lockdown area (2.45 ± 0.06).

### 4.5. The Confidence in Fighting COVID-19

**Figure 6:**
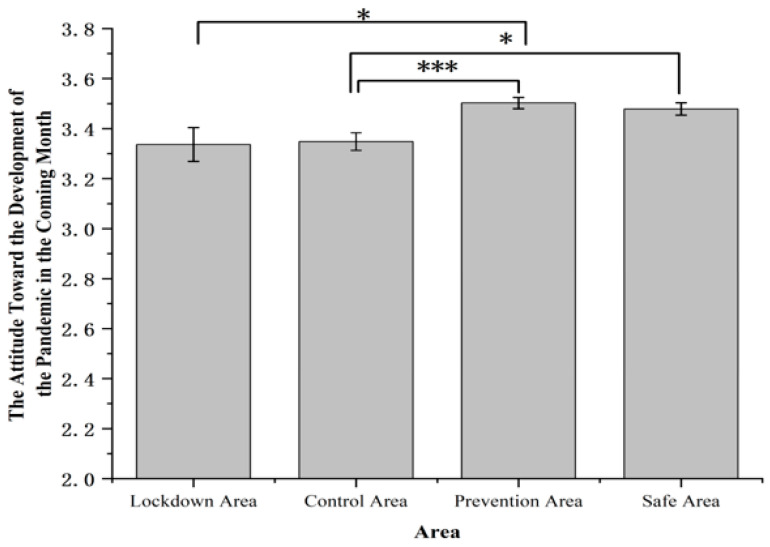
The attitude toward the development of the pandemic in the coming month. Note: The rating of attitudes toward the development of the pandemic in the coming month was measured on a 4-point scale (1 = much more severe, 2 = more severe, 3 = more relieve, 4 = much more relieve). Error bars represent standard errors. * *p* < 0.05, *** *p* < 0.001.

The attitude of the development of the pandemic in the coming month showed significant differences among four areas, *F* (3, 1631) = 6.52, *p* < 0.001, *η*^2^*_p_* = 0.012 (Figure 6). Respondents living in the lockdown area (3.34 ± 0.07) had a significantly more pessimistic view of the development of the pandemic compared with prevention area (3.50 ± 0.02, *p* < 0.05). Additionally, respondents living in the control area (3.35 ± 0.03) also had a significantly more pessimistic view of the development of the pandemic compared with the prevention area (3.50 ± 0.02, *p* < 0.001) and the safe area (3.48 ± 0.02, *p* < 0.05).

**Figure 7:**
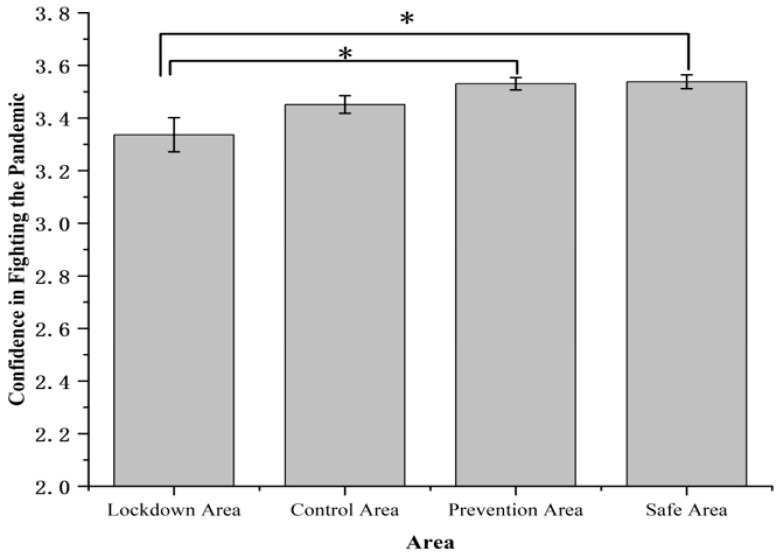
Confidence in fighting the pandemic. Note: The rating of confidence in fighting the pandemic was measured on a 4-point scale (1 = very negative, 2 = less negative 3 = more positive, 4 = very positive). Error bars represent standard errors. * *p* < 0.05.

Confidence in fighting the pandemic showed significant differences among four areas, *F* (3, 1631) = 4.44, *p* < 0.01, *η*^2^*_p_* = 0.008 (Figure 7). Participants living in the lockdown area (3.34 ± 0.06) had a significantly more pessimistic view of confidence in fighting the pandemic compared with the prevention area (3.53 ± 0.02, *p* < 0.05) and the safe area (3.54 ± 0.03, *p* < 0.05).

### 4.6. Correlation between Demographic Variables, Social Emotions, Concerns about the Pandemic, Perceived Efficiency, Transparency of the Government, and Confidence in Fighting the Pandemic

As shown in Table 3, the results showed that confidence in fighting the pandemic was positively associated with areas (*r* = 0.08, *p* < 0.01), optimism (*r* = 0.25, *p* < 0.001), calm (*r* = 0.18, *p* < 0.001), concerns about the pandemic (*r* = 0.16, *p* < 0.001), perceived efficiency (*r* = 0.35, *p* < 0.001), and transparency of the government in publishing pandemic-related information (*r* = 0.33, *p* < 0.001).

Confidence in fighting the pandemic was negatively associated with education (*r* = −0.06, *p* < 0.05; i.e., people with less education had more confidence in fighting the pandemic), worry (*r* = −0.19, *p* < 0.001), helplessness (*r* = −0.22, *p* < 0.001), fear (*r* = −0.21, *p* < 0.001), sadness (*r* = −0.19, *p* < 0.001), anger (*r* = −0.23, *p* < 0.001), and restlessness (*r* = −0.23, *p* < 0.001).

Sex, age, subjective SES, and time in reading or searching for pandemic-related information were not significantly associated with confidence in fighting the pandemic, all *ps* > 0.05. The Benjamini–Hochberg procedure (1995) was used to control the false positive rate for multiple comparisons.

### 4.7. Regression Analysis

The predictors in the regression analysis were chosen according to the results of the correlation of all variables (Table 3). The results of the three steps of hierarchical regression analysis are shown in Table 4. The first step of hierarchical regression analysis revealed that areas and education level were significant predictors of confidence in fighting the pandemic. These factors explained 1.3% of the variance in confidence fighting the pandemic, *F* (2, 1632) = 10.72, *p* < 0.001. Specifically, participants in the areas under less stringent control measures (β = 0.10, *p* < 0.001), or with lower education level (β = −0.06, *p* < 0.01), reported more confidence in fighting the pandemic.

After controlling for areas and education level of participants, the second step of the analysis (model 2) revealed that social emotions explained 9.3% of the variance in confidence fighting the pandemic, *F* (8, 1624) = 21.08, *p* < 0.001. Participants with a higher rating of optimism (β = 0.17, *p* < 0.001) or lower rating of anger (β = −0.11, *p* < 0.001) reported more confidence fighting the pandemic.

After controlling for areas, education level, and social emotions, the third step of the analysis (model 3) revealed that concerns about the pandemic, perceived efficiency, and transparency of the government in publishing pandemic-related information explained 13% of the variance in confidence fighting the pandemic, *F* (3, 1621) = 92.14, *p* < 0.001. Participants with more concerns about the pandemic (β = 0.09, *p* < 0.001), higher rating of the efficiency (β = 0.21, *p* < 0.001), or higher rating of the transparency of government in publishing pandemic-related information (β = 0.18, *p* < 0.001) reported more confidence fighting the pandemic.

## 5. Discussion

The present study investigated the differences in social emotions, concerns about the pandemic, perceived efficiency, transparency of the government in disseminating the pandemic-related information, and confidence in fighting the pandemic among people across four areas under different lockdown strategies, after a week-long lockdown of Shenzhen during COVID-19 pandemic. The results show that the positive emotion (i.e., calm), perceived efficiency of the government, and confidence in fighting the pandemic were lower in the areas under stringent control measures (lockdown or control areas), except that people’s concerns about the pandemic were lower in the control area than prevention area, whereas negative emotions (i.e., fear, worry, sadness) were higher in the lockdown area. Moreover, after controlling for areas and education level of participants, the emotion of optimism, peoples’ concerns about COVID-19, perceived efficiency, and transparency of the government in releasing COVID-19 relevant information positively predicted confidence in fighting the COVID-19 pandemic, while anger negatively predicted confidence in fighting COVID-19.

Specifically, compared to other areas (i.e., the control area, prevention area, and safe area), the emotion of calm was significantly lower in the lockdown area, whereas the emotions of worry, fear, and sadness were significantly higher in the lockdown area. On the one hand, previous studies found that people closer to the disaster have greater negative emotions [36]. On the other hand, these results were consistent with the findings of previous studies that quarantine and social distancing can influence public emotions and mental health [37,38,39]. First, though the control measures such as social distancing are beneficial to residents’ physical health and significantly reduced the infection rate, they have a negative impact on residents’ mental health, in the long term, and on their welling-being [7,40]. How to balance stringent restrictions and peoples’ fundamental need for social connections is a challenge. Second, maintaining positive emotions and reducing negative emotions are also important to perceived efficacy, adherence, and execution of control measures. Compared with the nationwide lockdown, local authorities were being encouraged to take precise prevention and control or differentiate isolation measures according to local conditions (e.g., the number of new cases), which may solve this contradiction to some extent [41]. Moreover, local governments and mental health institutions can provide intervention services both offline and online (e.g., 24-h hotlines) in a timely manner, besides supporting people with mental health information [42].

Residents’ concerns about the pandemic in the control area were lower than that in the prevention area. This was similar to the typhoon eye effect found in previous studies about the SARS or Wenchuan earthquake, in which people at the center of a risk event have lower risk perceptions of the event compared to those in areas outside the event [43,44]. This can be explained by cognitive dissonance theory [45,46]. That is, in order to avoid cognitive dissonance, residents in the control area changed their attitudes (for example, they consider the pandemic to be less of a concern) toward the pandemic when they cannot change the situation [45]. Other explanation might be that the residents in the control area were more likely to adapt to the situation under the pandemic, thus lowering the risk perception [37].

Perceived efficiency of the government in publishing pandemic-relevant information was lower in the lockdown area than that in the prevention or safe area. This result can be explained by the effect of emotion on time perception. Previous studies suggested that emotion affects time perception [47,48,49]. More specifically, this effect is modulated by individual differences, e.g., compared with non-anxious people, anxious individuals overestimated the duration of threatening stimuli [50]. Moreover, previous studies investigating the perceived boredom and perception of time during the quarantine under COVID-19 also found the perceived time longer compared with the pre-pandemic level [51,52]. In the present study, residents in the lockdown area who were more worried or fearful about the pandemic-relevant events (Figure 1) may overestimate the duration of the lockdown, thus having more requirements for the efficiency of the government’s actions in dealing with the pandemic.

The results showed that residents in the lockdown area are less confident compared with the control, prevention, or safe areas. More importantly, after controlling for areas and education level of participants, our study showed the emotion of optimism, concerns about the COVID-19 pandemic, perceived efficiency, and transparency of the government in releasing COVID-19 relevant information positively predicted confidence in fighting COVID-19, while anger negatively predicted confidence in fighting COVID-19. The positive role of the transparent communication of the government on the public trust and attitudes toward the control measures was also suggested in previous studies [53,54,55]. For example, research on COVID-19 pandemic lockdowns suggested that the perceived capability of the government positively affected public confidence and policy effectiveness [53]. The transparent communication of the government or health institutes can increase public trust, perceived risks of COVID-19, and encourage a positive attitude to the control measures [54]. These results suggested that it is necessary to improve efficiency and transparency in publishing pandemic-related information during the lockdown, which can promote public confidence, and further strengthen the implementation of control measures.

Some limitations of this study should be mentioned. First, this survey was cross-sectional and cannot make causal inferences about relationships between variables. Longitudinal studies could be used to examine social perceptions and attitudes during the pandemic. Second, the present study included 110 (6.8%) and 319 (19.6%) participants in the lockdown area and control area, respectively, which may limit the representativeness of the samples of these areas. Moreover, the present study contained more females (65.5%) than males (34.5%), and most of the samples (67.6%) were young (aged 18–29). These might limit the representativeness of the samples. According to the National Census, as of 1 November 2020, the population of Shenzhen was 17.56 million (National Bureau of Statistics of China), with 79.53% of the population aged 15–59 [56]. Though there were no details for the distribution of the age between 15 and 59 in the National Census, according to a census of 2017 (Shenzhen Municipal Working Committee on Women and Children), the median age of the population in Shenzhen is 31.95 years [57]. Thus, it is reasonable that our participants were generally young, and the population of old people was small. Third, the present study mainly utilized subjective self-assessment reports. This may have biased estimates. Future research can include objective and subjective indicators to more accurately assess people’s social emotions and perceptions.

The present study extended an understanding of the influence of the control measures on social emotions, concerns about the pandemic, as well as attitudes of residents toward the government, and sheds light on the measures to improve confidence in fighting the COVID-19 pandemic. These findings can also help the government to make better control measures. First, the government should consider people’s mental health besides the execution of the control measures. For example, when the government or authorities develops control measures, “tight–loose ambidexterity” should be considered, because appropriate looseness is associated with positive social emotions while holding stringent restrictions of social distancing [21]. In the longer term, it might contribute to the residents’ mental health during public health emergencies. Moreover, to minimize the psychological effect of the pandemic, the government should increase awareness of mental disorders and provide psychological interventions, especially to the people at high risk of psychological problems [58]. Second, the government should promote efficiency and transparency in releasing COVID-19-relevant information—for example, publishing information about the pandemic or public events in a timely manner, explaining the necessities to implement quarantine, taking swift responses to reduce negative emotions. With the enhanced positive emotions, these strategies can promote confidence in fighting COVID-19.

## 6. Conclusions

The present study investigated the factors influencing confidence in fighting the COVID-19 pandemic, including social emotions, concerns about the COVID-19 pandemic, perceived efficiency, and transparency of the government across four areas with different control measures imposed by the government of Shenzhen. We found that the tightness of the control measure was associated with increased negative emotions and decreased positive emotions, concerns about the pandemic, the perceived efficiency of the government, and confidence in fighting the pandemic. More importantly, social emotions, concerns about the COVID-19 pandemic, perceived efficiency, and transparency of the government can predict confidence in fighting COVID-19. These findings suggested that the government and communities make efforts at effective communication and find innovative approaches to keep social connections, reduce negative emotions, and enhance confidence in combating the pandemic.

## Figures and Tables

**Table 1:** Demographic characteristics (N = 1635).

	N	%
**Sex**		
*Female*	1071	65.5
*Male*	564	34.5
**Age**		
*18*–*29*	1106	67.6
*30*–*39*	357	21.8
*40*–*49*	116	7.1
*50*–*59*	49	3
*60 or older*	7	0.4
**Education**		
*Primary school*	9	0.6
*Middle school or Technical school*	42	2.6
*High school*	112	6.9
*College*	314	19.2
*Undergraduate or Bachelor’s degree*	964	59
*Master’s degree or above*	194	11.9
**Areas**		
*Lockdown Area*	111	6.8
*Control Area*	321	19.6
*Prevention Area*	648	39.6
*Safe Area*	555	33.9

**Table 2:** Social emotions during the pandemic control.

Variable	Mean	SE
Positive emotions	3.34	0.02
Optimism	3.34	0.02
Calm	3.35	0.03
Negative emotions	2.22	0.02
Worry	2.90	0.03
Helplessness	2.11	0.03
Fear	1.92	0.03
Sadness	2.11	0.03
Anger	1.89	0.03
Restlessness	2.36	0.03

**Table 3:** Correlation between demographic variables, social emotions, concerns about the pandemic, perceived efficiency, transparency of the government, and confidence in fighting the pandemic.

	1	2	3	4	5	6	7	8	9	10	11	12	13	14	15	16	17
1. Sex																	
2. Age	−0.04																
3. Areas	−0.08 **	0.07 **															
4. Education	−0.02	−0.10 ***	0.05														
5. Subjective SES	0.01	0.05	−0.01	0.24 ***													
6. Optimism	0.07 *	−0.08 **	0.05	0.00	0.16 ***												
7. Calm	0.09 ***	−0.03	0.06	0.07 *	0.15 ***	0.53 ***											
8. Worry	−0.07 *	−0.05	−0.07 *	0.03	−0.06	−0.36 ***	−0.38 ***										
9. Helplessness	−0.03	0.01	−0.06 *	−0.01	−0.07 *	−0.34 ***	−0.37 ***	0.59 ***									
10. Fear	−0.08 **	0.03	−0.04	−0.03	−0.07 **	−0.30 ***	−0.34 ***	0.55 ***	0.69 ***								
11. Sadness	−0.08 **	−0.04	−0.04	0.01	−0.05	−0.30 ***	−0.31 ***	0.49 ***	0.61 ***	0.64 ***							
12. Anger	−0.00	0.01	−0.03	0.03	−0.02	−0.26 ***	−0.30 ***	0.38 ***	0.51 ***	0.51 ***	0.57 ***						
13. Restlessness	−0.06	−0.03	−0.01	0.05	−0.07 **	−0.32 ***	−0.38 ***	0.46 ***	0.52 ***	0.50 ***	0.52 ***	0.64 ***					
14. Time in reading or searching for pandemic-related information	0.07 **	0.04	−0.02	−0.05	0.11 ***	0.05	−0.04	0.10 ***	0.14 ***	0.13 ***	0.13 ***	0.14 ***	0.11 ***				
15. Concerns about the pandemic	−0.01	0.09 ***	0.03	−0.04	0.06	0.05	−0.03	0.08 **	0.05	0.11 ***	0.08 **	0.06	0.06 *	0.39 ***			
16. Perceived efficiency of the government in publishing pandemic-related information	0.00	−0.00	0.04	−0.04	0.04	0.19 ***	0.17 ***	−0.09 ***	−0.09 ***	−0.06 *	−0.02	−0.07 **	−0.08 **	0.08 **	0.23 ***		
17. Perceived transparency of the government in publishing pandemic-related information	0.02	0.02	0.02	0.05	0.09 ***	0.15 ***	0.09 ***	−0.06	−0.09 ***	−0.08 **	−0.06 *	−0.13 ***	−0.09 ***	0.02	0.15 ***	0.46 ***	
18. Confidence in fighting the pandemic	−0.03	0.00	0.08 **	−0.06 *	0.04	0.25 ***	0.18 ***	−0.19 ***	−0.22 ***	−0.21 ***	−0.19 ***	−0.23 ***	−0.23 ***	−0.02	0.16 ***	0.35 ***	0.33 ***

Note: Benjamini–Hochberg procedure (1995) was used to control the false positive rate for multiple comparisons. * *p* < 0.05, ** *p* < 0.01, *** *p* < 0.001.

**Table 4:** Predicating the expectation about anti-COVID-19 in Hierarchical Regression Analyses.

Predictors	The Expectation about Anti-COVID-19					
	Model 1		Model 2		Model 3	
	β	SE	β	SE	β	SE
Areas	0.1 ***	0.09	0.08 ***	0.09	0.06 **	0.08
Education	−0.06 **	0.09	−0.06 **	0.09	−0.05 *	0.08
*Social emotions*						
Optimism			0.17 ***	0.09	0.10 ***	0.09
Calm			0.003	0.1	−0.01	0.09
Worry			−0.03	0.09	−0.04	0.08
Helplessness			−0.04	0.1	−0.01	0.09
Fear			−0.02	0.11	−0.04	0.1
Sadness			0.01	0.1	−0.03	0.09
Anger			−0.11 ***	0.1	−0.09 **	0.09
Restlessness			−0.05	0.09	−0.04	0.08
*Concerns about the pandemic, perceived efficiency, and transparency of the government*						
Concerns about the pandemic					0.09 ***	0.12
Perceived efficiency of the government in publishing pandemic-related information					0.21 ***	0.15
Perceived transparency of the government in publishing pandemic-related information					0.18 ***	0.15
ΔR^2^	0.013 ***		0.093 ***		0.13 ***	

Note. Areas: 1 = Lockdown Area, 2 = Control Area, 3 = Prevention area, 4 = Safe Area. Education: 1 = Primary school, 2 = Middle school or Technical school, 3 = High school, 4 = College, 5 = Undergraduate or Bachelor’s degree, 6 = Master’s degree and above. * *p* < 0.05, ** *p* < 0.01, *** *p* < 0.001.

## Data Availability

The data analyzed in the current study are available from the authors on reasonable request.
